# Combination Therapy Strategies for the Treatment of Malaria

**DOI:** 10.3390/molecules24193601

**Published:** 2019-10-07

**Authors:** Sibusiso Alven, Blessing Aderibigbe

**Affiliations:** Department of Chemistry University of Fort Hare, Alice Campus, Alice, Eastern Cape 5700, South Africa; 201214199@ufh.ac.za

**Keywords:** malaria, antimalarial drugs, combination therapy, hybrid compounds, polymer-based carriers

## Abstract

Malaria is a vector- and blood-borne infection that is responsible for a large number of deaths around the world. Most of the currently used antimalarial therapeutics suffer from drug resistance. The other limitations associated with the currently used antimalarial drugs are poor drug bioavailability, drug toxicity, and poor water solubility. Combination therapy is one of the best approaches that is currently used to treat malaria, whereby two or more therapeutic agents are combined. Different combination therapy strategies are used to overcome the aforementioned limitations. This review article reports two strategies of combination therapy; the incorporation of two or more antimalarials into polymer-based carriers and hybrid compounds designed by hybridization of two antimalarial pharmacophores.

## 1. Introduction

Malaria has remained a health burden around the world for decades, especially in the tropical regions, despite the several strategies that have been developed to combat the disease [1]. Malaria is a disease caused by the parasitic protozoan *Plasmodium* which is transferred by an infected female *Anopheles* mosquito [2]. The most virulent *Plasmodium* species among the five species that are accountable for the high rate of death in humans is *P. falciparum* [3]. The Malaria World Report indicated that malaria cases increased to 219 million in 2017 compared to 217 million cases reported in 2016. The number of malaria deaths reported in 2017 was 435,000 [4].

The major reason for the great mortality caused by malaria infections globally is drug resistance [5]. Chloroquine, the most active antimalarial, suffers from drug resistance [6]. Combination therapy is a promising approach that is currently employed to overcome drug resistance [7,8]. Combination therapies involve the design of hybrid compounds, where two or more bioactive agents are combined together [9] via selected functionalities, and the incorporation of drugs into polymer-based carriers [10]. Hybrid compounds are synthesized by hybridization of pharmacophores via selected functionalities. Due to the efficacy of combining two or more antimalarial drugs, WHO has approved combination therapies, classified as artemisinin-based combination therapies (ACTs) and nonartemisinin-based therapies [11].

Polymer-based carriers are nanocarriers used as vehicles to deliver therapeutic agents to the target biological environment [12]. There are several forms of polymer-based carriers, such as polymer–drug conjugates [13], polymer nano- and microcapsules [14], in-situ gels, hydrogels and nano gels [15], dendrimers, and micelles [16]. These polymer-based carriers loaded with therapeutic agents display unique properties causing them to be good potential systems to combat several chronic diseases, such as bone diseases, brain diseases, neurodegenerative diseases, cancer, and infectious diseases, such as Human Immunodeficiency Virus (HIV) and malaria [17]. This review article reports the in vitro and in vivo therapeutic outcomes of the hybrid compounds and polymer-based carriers containing antimalarial drugs. The prepared antimalarial drug-based hybrid compounds that will be reviewed in this article are those that have been reported in the last five years (2014–2019).

## 2. Classification of Antimalarial Drugs 

**Figure 1:**
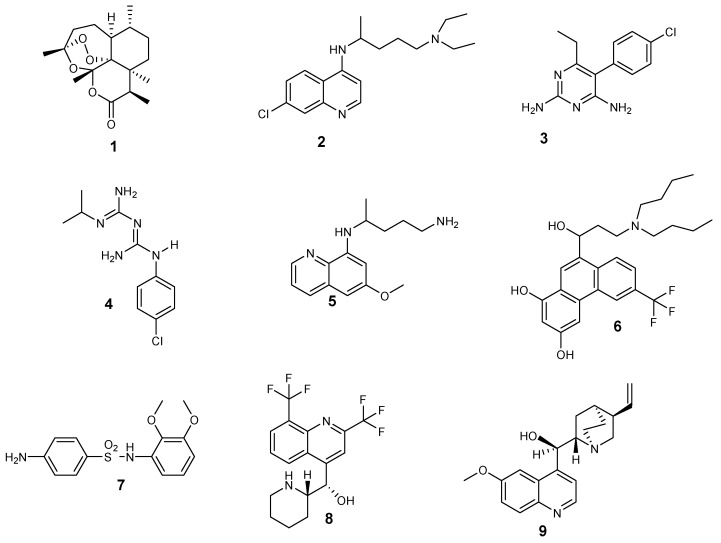
Structures of some antimalarial drugs.

Different types of antimalarial agents are classified on the basis of their chemical structure and antiplasmodial activity in the malaria life cycle. According to the antiplasmodial activity, five classes of antimalarial agents have been categorized [18]: gametocidal, prophylaxis, blood schizonticides, tissue schizonticides, and sporontocides, as shown in Figure 1. Gametocytocides are administered to inhibit the transfer of malaria from an infected person to uninfected female *Anopheles* mosquito. Gametocytocides destroy female and male gametocytes of the parasites in the blood stage of the malaria life cycle. Some examples of gametocytocides include artemisinin **1** and chloroquine **2**. Prophylactic antimalarial bioactives are administered for the prevention of malaria infections in people who are travelling from nonmalaria countries to malaria-endemic countries, especially travelers with low immune function. Prophylactic drugs include pyrimethamine **3**, proguanil **4**, and primaquine **5** [19].

Blood schizonticides are utilized to disrupt asexual erythrocyte forms of the *Plasmodium* parasites and stop the early symptoms of malaria. Some examples of blood schizonticides include halofantrine **6**, sulfadoxine **7**, mefloquine **8**, and quinine **9** [20]. Tissue schizonticides are employed to prevent the relapse of *P. ovale* and *P. vivax* parasites caused by hypnozoites in the liver stage of the plasmodium life cycle [21]. Pyrimethamine **3** and primaquine **5** are examples of tissue schizonticides. Sporontocidal antimalarial drugs inhibit the development of oocytes in the parasites in the mosquito stage of the *Plasmodium* life cycle, thereby inhibiting the transmission of the disease. Sporontocidal drugs include primaquine **5** and pyrimethamine **3**.

**Figure 2:**
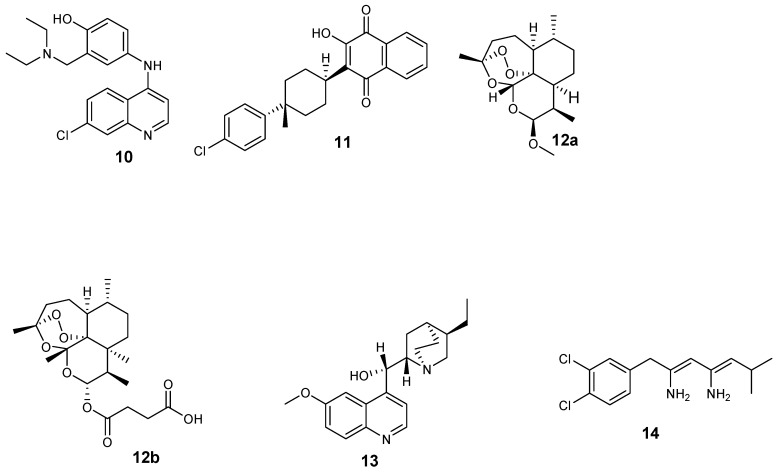
Structure of antimalarial drugs classified based on their chemical structures.

Antimalarials are also classified based on their structures, as shown in Figure 2 [22]: 4-aminoquinolines (amodiaquine **10** and chloroquine **2**); 8-aminoquinolines (primaquine **5**); hydroxynaphthoquinones (atovaquone **11**); artemisinin derivatives (Artemether **12a** and artesunate **12b**); diaminopyrimidines (pyrimethamine **3**); quinolines-based cinchona alkaloids (quinine **9** and quinidine **13**); 4-quinolinemethanols (mefloquine **8**); biguanides (proguanil **4** and chloroproguanil **14**); and sulfonamides (sulfadoxine **7**).

## 3. Combination Therapy Strategies

### 3.1. Antimalarial Hybrid Compounds

Antimalarial-based hybrid compounds are categorized based on the type of pharmacophores that are hybridized together. Most of the currently reported antimalarial hybrid compounds can be classified as artemisinin-based hybrid compounds and nonartemisinin hybrid compounds.

#### 3.1.1. Artemisinin-Based Hybrid Compounds

**Figure 3:**
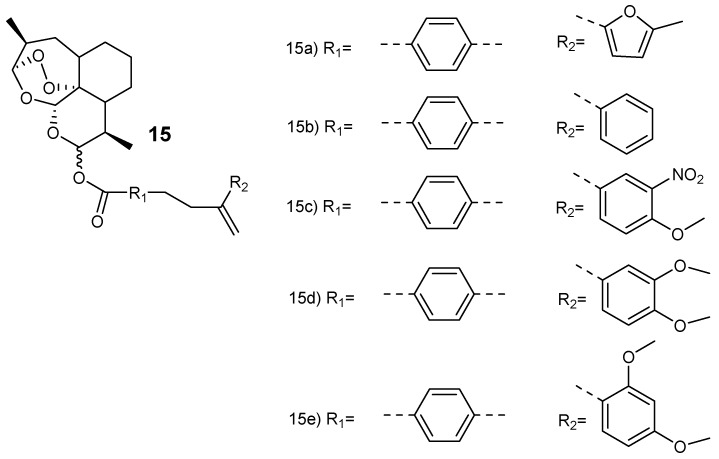
Dihydroartemisinyl-chalcone hybrid compounds **15**.

Smit et al. synthesized hybrid compounds via esterification of dihydroartemisinin with chalcones to produce dihydroartemisinyl-chalcone esters (**15a**–**e**) (Figure 3) [23]. The hybrids were assessed against chloroquine-resistant (W2) and chloroquine-sensitive (3D7) strains in vitro. They were potent against both strains with 50% inhibitory concentration (IC_50_) values ranging between 1.5–11 nM and selectivity index (SI) values above 5800. The hybrids with oxygen-containing aryl rings in the chalcone (**15a**, **15d** and **15e**) displayed similar antimalarial activity as dihydroartemisinin. However, they were two- to three-fold more effective when compared to artesunate against the W2 and 3D7 strains of the plasmodium parasites. The thermogravimetric analysis further revealed that the hybrids were thermally stable when compared to dihydroartemisinin, thereby favoring high-temperature storage environments which are peculiar to the tropical malaria-endemic regions [23]. 

**Figure 4:**
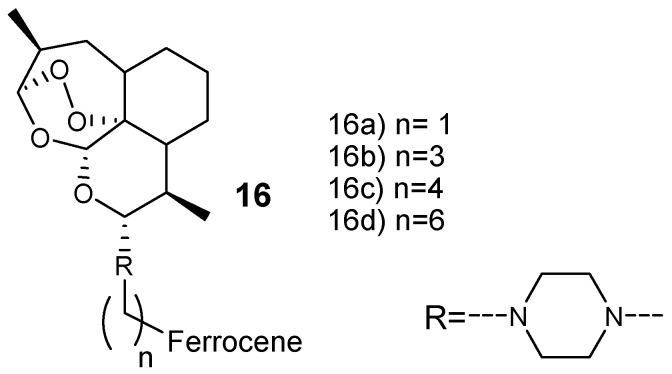
Artemisinin–ferrocene hybrid compounds (**16**).

Lange et al. prepared new artemisinin-based hybrid compounds **16** by attaching ferrocene with dihydroartemisinin via piperazine linker, as shown in Figure 4. They were evaluated against chloroquine-resistant W2 and K1 in vitro, and chloroquine-sensitive NF54 strains of *P. falciparum* parasites [24]. These hybrids exhibited antimalarial efficacy in the minimum range of nM and the most potent hybrid compound was hybrid **16a** with an IC_50_ value of 3.2 nM against *P. falciparum* W2 and 2.79 nM against *P. falciparum* K1. The overall resistance indices of the hybrid compounds was in the range of 0.5–0.7, suggesting that these hybrid compounds have a low potential for cross-resistance. Furthermore, the selectivity indices of the hybrid compounds were greater than 9000 revealing a significant high selectivity toward *Plasmodium* parasite when compared to the mammalian cells [24]. 

**Figure 5:**
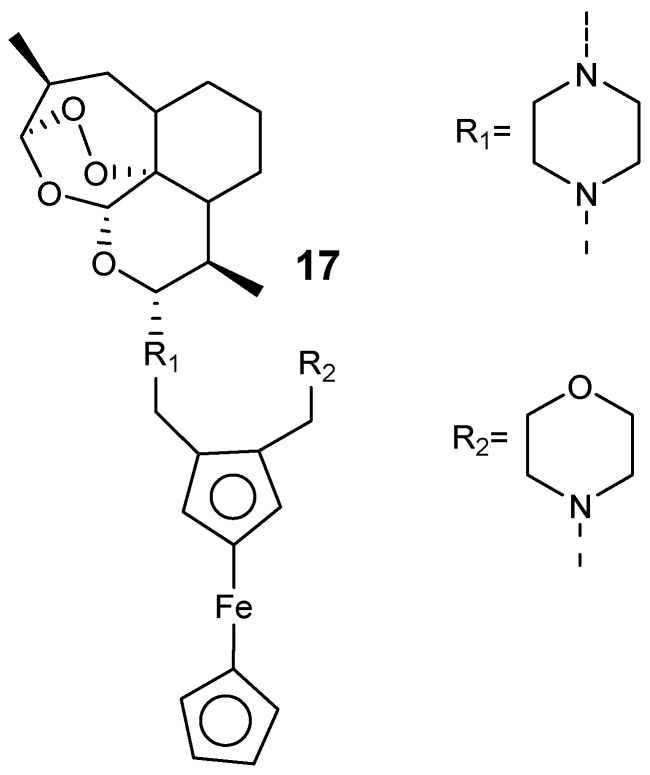
Artemisinin–ferrocene hybrid compounds (**17**).

Furthermore, Lange et al. prepared hybrid compounds by incorporating a 1,2-disubstitutedferrocene derivative into the piperazine artemisinin derivative C10 via a piperazine linker, as shown in Figure 5 [25]. The most active hybrid compound was hybrid **17** with IC_50_ values of 1.4 nM against *P. falciparum* W2 and 0.86 nM against *P. falciparum* K1. The resistance indices of **17** was 0.2 when compared to artemisinins, 0.6 for dihydroartemisinin, 1.3 for artesunate, and 4.8 for artemether. These hybrids were potent against *P. falciparum* NF54 gametocytes at the initial and final blood-stage with the inhibition concentration of more than 86% at 1 µM. 

**Figure 6:**
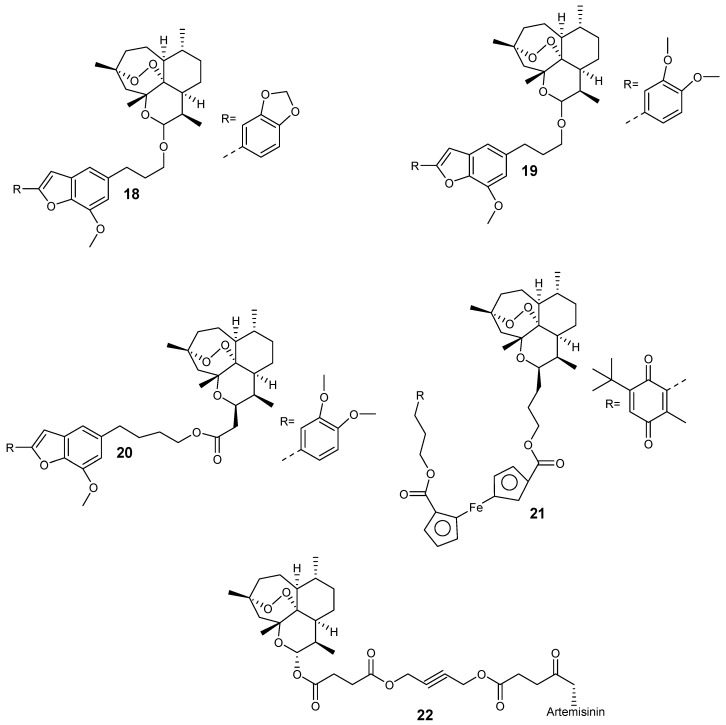
Artemisinin-based hybrid compounds.

Capci et al. synthesized new hybrid compounds (Figure 6) from artemisinin and selected natural products [26]. These hybrids displayed good antiplasmodial efficacy when compared to their parental drugs, with half-maximal effective concentration (EC_50_) values in the nanomolar range. In particular, the EC_50_ value of hybrid **18** was 28.3 nM, while for compounds **19** and **20**, the EC_50_ was 13.3 nM and 11.86 nM, respectively. Most significantly, compound **21** displayed an EC_50_ value of 6.5 nM, out-performing the reference antimalarial drug, chloroquine, which exhibited an EC_50_ value of 9.8 nM. The hybrid-dimer **22** (EC_50_ = 3.8 nM) was two-fold more active than artesunic acid (EC_50_ = 9.7 nM) and chloroquine (EC_50_ = 9.8 nM).

**Figure 7:**
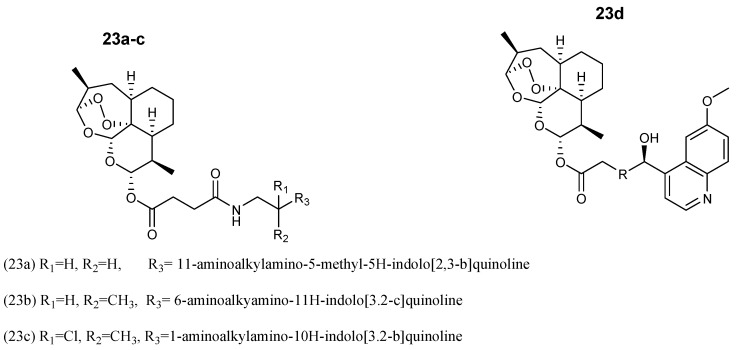
Structure of the artesunate–quinoline-based hybrid compound (**23**).

Wang et al. prepared the artesunate–quinoline hybrid **23a**–**c** (Figure 7) with improved antimalarial activity when compared to the free therapeutic agent. They were synthesized by the reaction of artesunate with quinoline derivatives in the presence of EDCI and HOBt at room temperature. The IC_50_ value of **23** was 0.42 nM and 0.45 nM against strains K1 and NF 54 strains, respectively with a resistance index of 0.93. The compound exhibited a significant reduction in parasitemia over a period of four days with an efficacy of 89.6%, and a mean existence period of 7.7 days in vivo [27]. Furthermore, Walsh et al. hybridized dihydroartemisinin with quinine carboxylic acid via ester linkage **23d** [28]. The hybrid compound displayed superior antimalarial activity when compared to the individual parent drugs, artemisinin, quinine alone, or a 1:1 mixture of both parent drugs, confirming that the artemisinin and quinine moieties were well-conserved.

**Figure 8:**
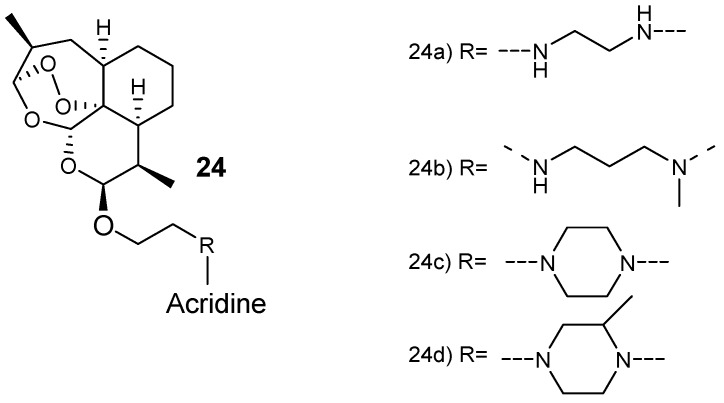
Structure of artemisinin–acridine hybrid compounds **24**.

Joubert et al. synthesized artemisinin–acridine-based hybrid compounds **24** (Figure 8) [29]. The hybrids were synthesized via a microwave-assisted radiation method by reacting artemisinin and acridine pharmacophores using an aminoethyl ether linker. These hybrid compounds exhibited antiplasmodial activity against both sensitive chloroquine and chloroquine-resistant (Dd2) strains with a higher selective toxicity to the parasitic cells. Hybrid compound **24a**, having an ethylenediamine linker, was the most effective derivative, seven-fold more active than the clinically used antimalarial drug, chloroquine, against both the gametocytocidal strain Dd2 and NF54 strain of *P. falciparum*, with very selective action against the parasitic cells [29]. 

#### 3.1.2. Nonartemisinin-Based Hybrid Compounds

There are several antimalarial hybrid compounds that were not synthesized from artemisinin derivatives and they have been reported to be active against *Plasmodium* parasite depending on the stages of the *Plasmodium* life cycle. This section will be focused on quinoline-based and ferrocene-based hybrids.

##### Quinoline-Based Hybrid Compounds

**Figure 9:**
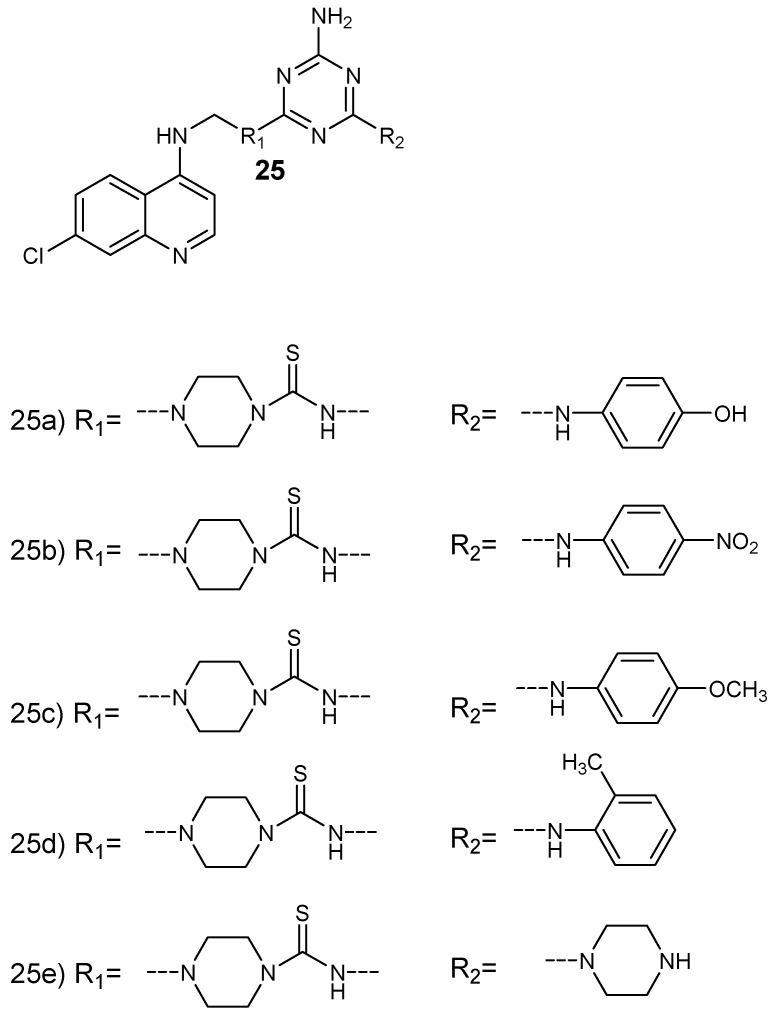
Structure of 4-aminoquinoline-triazine hybrid compounds **25a**–**e**.

Bhat et al. prepared a series of 4-aminoquinoline-based hybrid compounds **25** by incorporating 4-aminoquinoline with 1,3,5- triazine (Figure 9) [30]. The hybrid compounds were isolated in good yields in the range of 46–73%. In vitro antiplasmodial evaluation against chloroquine-sensitive (3D7) and chloroquine-resistant (RKL-2) strains of *P. falciparum* was performed. The synthesized hybrids containing aromatic group with fluoro, chloro, and morpholino functional groups exhibited antiplasmodial activity against 3D7 similar to chloroquine but higher than proguanil. The two hybrid compounds, **25a** and **25c**, showed good antiplasmodial efficacy against both *P. falciparum* strains. The antiplasmodial activity of these hybrids were further investigated by docking studies on quadruple and wild type dihydrofolate reductase-thymidylate synthase of *P. falciparum* (*pf*-DHFR-TS). The hybrids containing 1,3,5-traizine and 4-aminoquinoline did not exhibit potent antimalarial activity. These compounds inhibited hemozoin formation and some were more active when compared to chloroquine in inhibiting beta-hematin production. The presence of aromatic lipophilic side chain also contributed to the antimalarial activity of the hybrids when compared to the parent drug, chloroquine. There are other researchers who synthesized similar 4-aminoquinoline-triazine hybrid compounds and they displayed significantly antimalarial activity [31,32]. 

**Figure 10:**
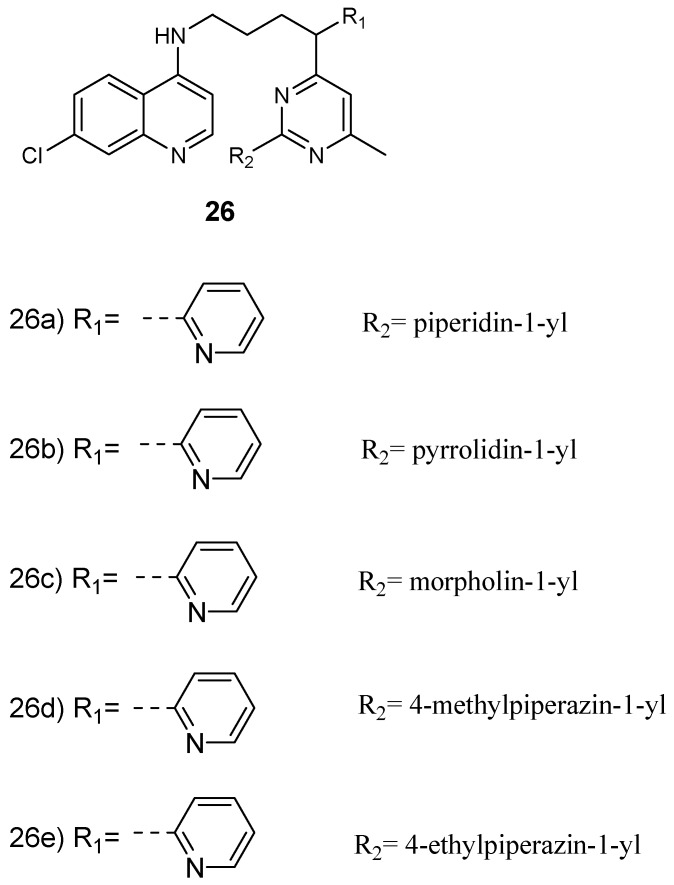
Structure of 4-aminoquinoline-pyrimidine hybrid compounds.

Maurya et al. prepared and examined the in vitro antiplasmodial efficacy of 4-aminoquinoline-pyrimidine hybrid compounds **26** (Figure 10) against both *P. falciparum* chloroquine-sensitive (D6) and chloroquine-resistant (W2) strains [33]. Most of the hybrids exhibited good in vitro antimalarial efficacy when compared to the standard drug, chloroquine, against both *P. falciparum* strains, with IC_50_ values in the range 0.0189–0.945 µM. The most active hybrid **26d** was further studied for heme binding and heme was found to be a target site of the hybrid compound. In addition, docking studies on *Pf*-DHFR displayed interesting binding interactions in the active position [33,34]. In another report, 4-aminoquinoline-pyrimidine hybrid compounds were isolated in good yields in the range of 82–92%. The hybrid compounds were four-fold more active when compared to chloroquine with an IC_50_ value of 56 nM [35]. The presence of *m*-nitrophenyl substituent at C-4 of the pyrimidine ring in the hybrid compound exhibited significant antimalarial activity. In hybrid molecules with a four-methylene spacer, antimalarial activity was high when compared to hybrids containing three-methylene spacer. The introduction of an aliphatic linker which was flexible with a piperazinyl linker resulted in the total loss of the antimalarial activity. Factors such as the steric restrictions caused by the piperazinyl linker hindered face-to-face stacking of the quinoline moiety with the heme, thereby hindering heme polymerization to hemozoin [35]. Cytotoxicity of the hybrid compounds against murine leukemia cells (L1210), human T-lymphocyte cells (CEM), and human cervix carcinoma cells (HeLa) revealed that most of these hybrids were more cytotoxic when compared to chloroquine. 4-aminoquinoline-piperonyl-pyrimidine hybrid compounds synthesized by Kholiya and coworkers were effective antimalarials with IC_50_ values in the range of 0.02–5.16 µM. Some of the hybrids were eight-fold more active when compared to chloroquine [36]. The hybrid in which the chloro group on the pyrimidine was replaced with 4-ethyl piperazine was more active than amino-substituted hybrids. Hybrids with a pyrimidine ring attached at the 4^th^ position of the 4-aminoquinoline-piperonyl intermediates exhibited enhanced antimalarial activity when compared to compounds with the pyrimidine ring attached to the second position of the 4-aminoquinoline-piperonyl intermediates. Increasing the diamine spacer to three carbon atoms decreased the antimalarial activity of the hybrids. Hybrids containing ethylene diamine linkers were significantly active against strains of *P. falciparum* with IC_50_ values in the range of 0.05–0.29 μM against resistant strain and 0.02–0.05 μM against sensitive strain [36].

**Figure 11:**
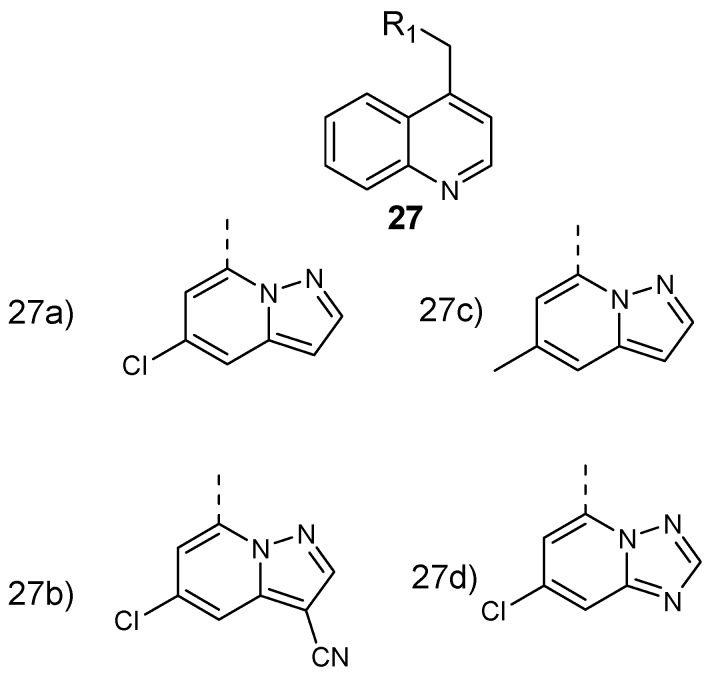
Structure of 8-aminoquinoline–pyrazolopyrimidine hybrid compounds **27a**–**d**.

Murugan et al. formulated and performed in vitro evaluation on 8-aminoquinoline-pyrazolopyrimidine hybrid compounds **27** (Figure 11) against both resistant strain (*Pf*-K1) and wild type (*Pf*-NF54) *Plasmodium falciparum*. These hybrid compounds exhibited potent antiplasmodial efficacy in a nanomolar range with IC_50_ values between 5 and 10 nM. The structure–activity relationship evaluation of the synthesized compounds showed that 8-aminoquinoline and the pyrazolopyrimidine ring are significant for their antiplasmodial activity against *P. falciparum* [37]. Converting the triazolopyrimidine core into a pyrazolopyrimidine ring resulted in a 100-fold improvement of antimalarial activity. Structural modification of the pyrazolopyrimidine ring at the C-5 position and the presence of electron withdrawing groups at the C-3 position retained the potency of the hybrid molecules. The introduction of a bulky group at position C-3 decreased the antiplasmodial activity of the compound [37].

**Figure 12:**
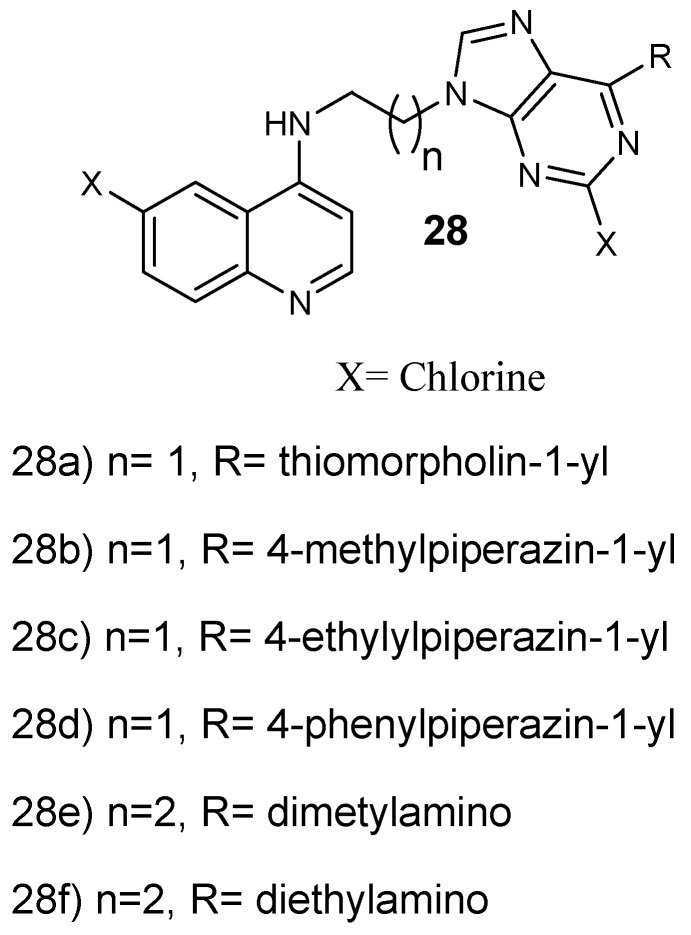
Structure of 4-aminoquinoline-purine hybrid compounds **28a**–**f**.

Reddy et al. prepared and performed antiplasmodial evaluation against *P. falciparum* CQ-resistant and CQ-sensitive strains on 4-aminoquinoline-purine hybrid compounds **27a**–**d** (Figure 12). These hybrids exhibited very good antiplasmodial activity against both strains, especially hybrid **28c** with up to six-fold greater activity compared to chloroquine, displaying an IC_50_ value of 0.08 µM. Most of the hybrids were noncytotoxic towards mammalian cells up to 11.86 µM [38]. Increasing the length of the spacer between the purine and aminoquinoline scaffold from two to three carbons decreased the antimalarial activity. However, hybrids substituted with 1-ethyl piperazines and 1-methyl, containing an ethylenediamine linker and a 1,3-propylenediamine linker exhibited a potent antiplasmodial activity. Hybrids with 1-phenyl piperazine substitutions exhibited reduced antimalarial activity [38]. Capela and coworkers synthesized endoperoxide–8-aminoquinoline hybrid compounds. These hybrids were active against the intra-erythrocytic *P. falciparum* W2 strain with EC_50_ values in the nM range. Furthermore, the hybrids showed antiplasmodial efficacy against *P. berghei* parasite at the liver stage with the EC_50_ ranging in low µM values [39]. Hybrid compounds with an amide linker between the two pharmacophores were very active, with IC_50_ values of 2 mM. Hybrid compounds lacking a linker between the two pharmacophores did not display significant liver stage activity, revealing that the linker contributes to the efficacy of the hybrid molecules against the liver stage parasites. The hybrids with either a heteroaryl or aryl functional group at the C-5 position of the 8-aminoquinoline moiety were effective as antiplasmodial agents. These hybrids inhibited the formation of intra-erythrocytic forms of *P. falciparum* and displayed low cytotoxicity against mammalian cells in vitro. The hybrids were found to be an interesting approach to eliminate intra- and exoerythrocytic parasites [39].

**Figure 13:**
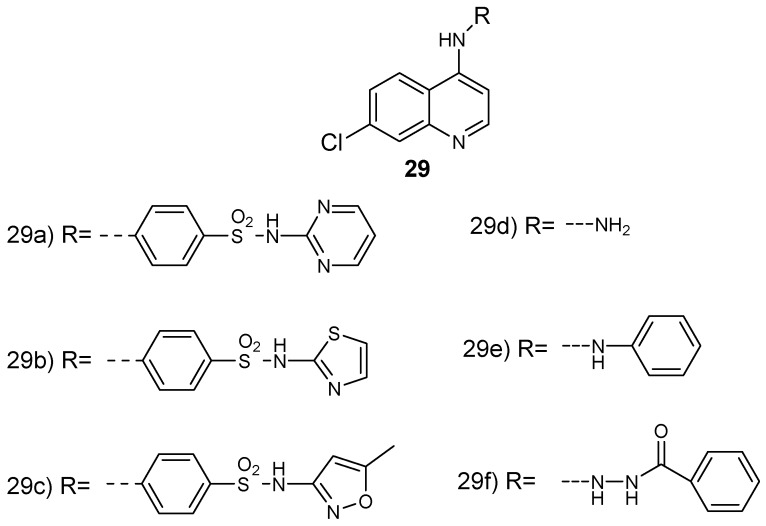
4-aminoquinoline–sulfonamide and hydrazine hybrids **29a**–**f**.

Soares et al. prepared quinoline-based hybrid compounds by combining aminoquinoline with sulfonamide **29a**–**c** and hydrazine **29d**–**f** (Figure 13) and they performed in vivo and in vitro antiplasmodial studies against *P. falciparum* chloroquine-resistant and chloroquine-sensitive strains and infected *P. berghei* (NK65) mice, respectively; and cytotoxicity studies against HeLa and HepG2 cell lines [40]. These hybrids displayed no cytotoxic effect against HeLa and HepG2 cell lines when evaluated up to a concentration of 100 µg/mL. The quinoline-hydrazine hybrids showed low cytotoxicity against the cell lines and exhibited good antimalarial efficacy against *P. falciparum* chloroquine-sensitive and chloroquine-resistant strains. Hybrid **29d** displayed antimalarial activity against blood parasites similar to chloroquine. Significantly, hybrid **29d** exhibited the best lipophilic efficiency (LipE) value of 4.84 when compared to those examined in vivo [40]. The hydrazine-derivative molecule **29d** was active in vivo against the mouse model of *P. berghei* infection. The compound displayed a significant reduction of blood parasites to levels superior to the clinically used drug, chloroquine, at a dose of 10 mg/mL.

**Figure 14:**
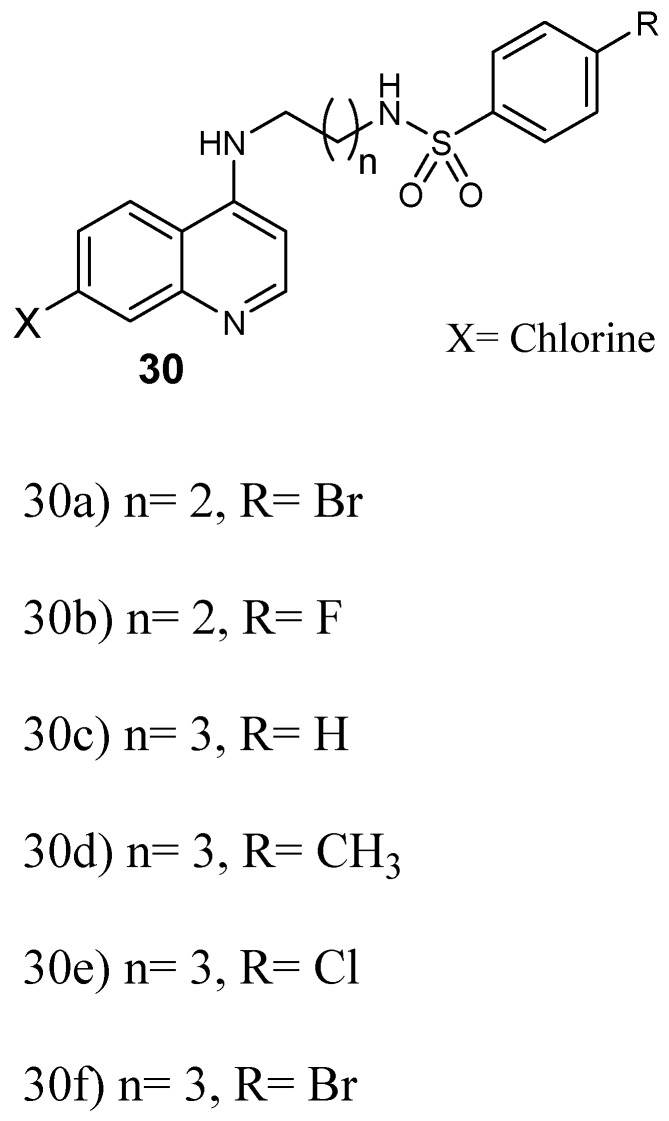
Structure of quinoline–sulfonamide hybrids **30a–f**.

Furthermore, Pinheiro and coworkers prepared quinoline–sulfonamide hybrids **30** (Figure 14) and they were assayed against *P. falciparum* [41]. The antiplasmodial screening of the hybrids revealed that the compounds displayed high in vitro schizonticidal blood efficacy with IC_50_ values in the micromolar range between 0.05 and 1.63 µM against CQ-resistant W2, whereby some of them displayed IC_50_ values in the range of 0.05–0.40 µM, lower than chloroquine and sulfadoxine. Hybrid **30e** displayed good antiplasmodial activity in vivo when compared to others and it was considered for further evaluation as an antimalarial agent against CQ-resistant plasmodium parasite [41]. Hybrids with three and four methylene group linkers exhibited greater activity against *P. falciparum* when compared to chloroquine. Hybrids with two methylene groups were not as effective as chloroquine. However, compounds with four methylene groups as linker were the most potent, with IC_50_ values in the range of 0.05–0.15 μM. Hybrids that did not contain a substituent on the arylsulfonamide group exhibited low antimalarial activity. The presence of a substituent at the 4-position of the arylsulfonamide group enhanced the efficacy of the hybrid compound [41].

Barteselli et al. synthesized novel indeno[2,1-c]quinolines hybrid compounds which were screened against chloroquine-resistant and chloroquine-resistant *P. falciparum* strains in vitro [42]. Most of these hybrids displayed moderate antimalarial activity against *P. falciparum* strains with IC_50_ values in the range of 0.24–6.9 µM. Some of the synthesized hybrids were found to be 1.2–1.3-fold more active compared to chloroquine against a chloroquine-resistant strain of *P. falciparum*. The resistance index of the hybrids was low, in the range of 0.3–1.6, indicating that the hybrids do not exhibit the same resistance mechanisms of chloroquine. The presence of the chlorine in position 2 or 3 of the indenoquinoline nucleus did not play any vital role in the antimalarial activity of the hybrid compounds. The presence of piperidinoethylamino and methylpiperazinoethylamino moieties improved the antimalarial efficacy of the hybrids, whereas the presence of 2-pyridinyl-4-piperazinyl and morpholinoethylamino moieties resulted in hybrid molecules which were not potent. The presence of an aminoguanidine functional group also enhanced the antimalarial activity of the hybrid molecules [42]. In addition, there are several recent reports of synthesized quinoline-based hybrids that exhibited promising therapeutic outcomes, especially potent antimalarial efficacy, such as trifloromethylquinoline hybrids [43], primaquine- and chloroquine-quinoxaline 1,4-di-*N*-oxide hybrids [44], 1,3-dioxoisoindoline-4-aminoquinolines [45], and atorvastatin–quinoline hybrid compounds [46]. Factors such as presence of the quinoline-amine group between the trifluoromethylquinoline ring and the water insoluble region enhanced the antimalarial activity of the hybrid molecules. Hybrid molecules with tertiary amines were rigid, which inhibited their capability to interrelate with the biomacromolecule receptor. The hybrid molecule containing a 3-methyl-1,2,4-triazole substituent exhibited good antimalarial activity of (IC_50_ = 0.083 µM) and it was three-fold more potent than chloroquine [45]. Primaquine hybrids exhibited low antimalarial activity in the erythrocytic stage resulting from the modest blood schizonticidal activity of primaquine and the absence of a basic amino group. The chloroquine-based hybrids were less active blood schizonticidal drugs against 3D7 and FCR-3 strains. The poor antimalarial activity of primaquine-based hybrids is due to the resistance-reversing effect of primaquine. However, the substitution at position 7 with a chlorine group increased in the blood stage activity and enhanced the antimalarial activity [44]. 1,3-dioxoisoindoline-4-aminoquinolines hybrid compounds’ antimalarial activity increased with an increase in the chain length, which was significant in glycyl and *β*-alanyl bonded compounds [45]. The presence of butyric acid and fluoro substituents at the C-5 position of dioxoisoindolines reduced the antiplasmodial activity of the hybrid compound. The presence of *β*-alanine at shorter chain lengths and secondary amines, such as diethylamine, morpholine, and 2-hydroxyethylpiperazine at C-5 of dioxoisoindoline, enhanced the basicity of the hybrid, thereby increasing the drug accumulation in the acidic digestive vacuole of the *Plasmodium* parasite. The presence of alanine between the amide linkage and dioxoisoindoline core among hybrids enhanced the antiplasmodial activities when compared to chloroquine [44]. Quinoline–sulfonamide hybrids composed of a 7-chloroquinoline moiety attached by a linker group to arylsulfonamide moieties with four methylene groups as linkers were effective against *P. falciparum*. The antimalarial efficacy of the hybrid compounds increased with increase in the length of the carbon chain [46].

##### Ferrocene-Based Hybrid Compounds

**Figure 15:**
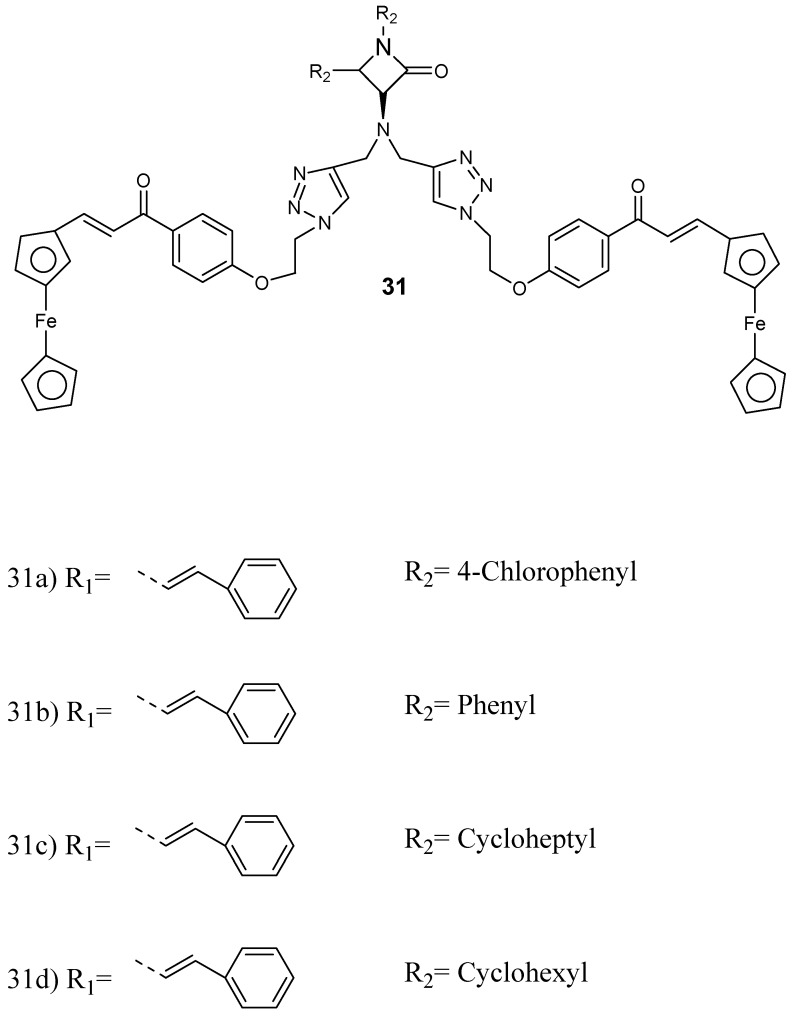
Structure of ferrocenylchalcone–b-lactam hybrid compounds **31a**–**d**.

Kumar et al. prepared and performed in vitro evaluation of a series of ferrocenylchalcone-β-lactam hybrid compounds **31** (Figure 15) against *P. falciparum* chloroquine-resistant (W2) and chloroquine-sensitive (3D7) strains [47]. The synthesized conjugates were not very active when compared to chloroquine. However, the presence of ferrocene moiety enhanced the antiplasmodial efficacy of the *β*-lactam nucleus. The antiplasmodial activity of the hybrid was influenced by the property of substituent at *N*−1 position of *β*-lactam. The length of the alkyl chain, which acted as the linker, and the presence of bis- and mono-ferrocenylchalcone did not influence the antimalarial efficacy of the conjugates. The hybrids **31c** and **31d** featuring an *N*-cyclohexyl substituent were the most active against *P. falciparum* W2 strain, showing IC_50_ values of 2.43 and 2.36 mM, respectively, and they were noncytotoxic when compared to other hybrids.

**Figure 16:**
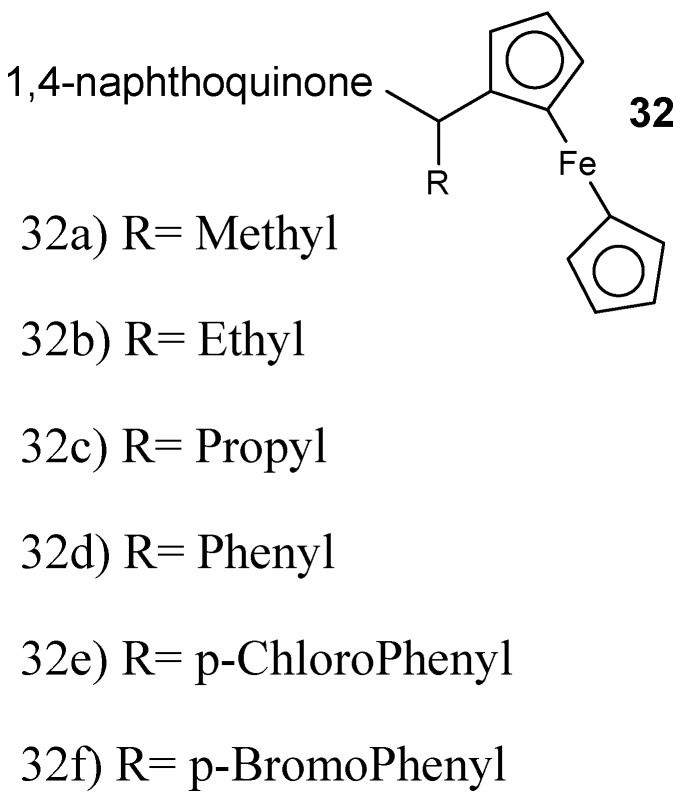
Structure of 3-ferrocenylmethyl-2-hydroxy-1,4-naphthoquinone **32a**–**f**.

Raj et al. described the synthesis 7-chloroquinoline-ferrocenylchalcone and 7-chloroquinoline-chalcone hybrid compounds followed by in vitro antimalarial analysis against the chloroquine-resistant *P. falciparum* (W2) strain. The antiplasmodial efficacy of 7-chloroquinoline-ferrocenylchalcone hybrid compounds was low when compared to their analogous simple chalcone conjugates. The antiplasmodial activity of these hybrids was improved by the presence of elongated alkyl chain and methoxy substituent at the para position of another ring on chalcones displaying an IC_50_ value of 17.8 nM [48]. The conjugate with propyl linker was very potent with IC_50_ of 73.4 nM. Increasing the chain length decreased the activity of the conjugates. The presence of a methoxy substituent enhanced the antimalarial efficacy of the conjugates, resulting in IC_50_ values in the range of 35.5–378 nM. The cytotoxicity studies showed that these hybrid compounds are noncytotoxic against mammalian cells and therefore had selectivity for inhibition of *P. falciparum* [48]. García-Barrantes and coworkers designed novel hybrid compounds **32** (Figure 16) synthesized by a 3-(ferrocenylmethyl)-1,4-naphthoquinone and they were evaluated in vitro against chloroquine- sensitive and chloroquine-resistant *P. falciparum* strains [49].

Ferroquine (FQ) is an analogue of chloroquine in which the ferrocene molecule is covalently linked to 4-aminoquinoline and a basic alkylamine [50]. Phase 2 clinical assessment showed that ferroquine is effective and safe against a multiresistant parasite and the chloroquine-resistant strains when used alone or in combination with artesunate. Its minimum and half-life inhibitory concentration is more than three weeks. It is not affected by food and it is well-tolerated up to 1600 mg in a single dose and at 800 mg for repeated dose. All these results show that FQ has tremendous potential to be utilized in clinics [50]. Ferroquine has the capability to overcome the chloroquine resistance problem. The lipophilicity of ferroquine is log *D* = −0.77 when compared to chloroquine which is log *D* = −1.2. Ferroquine also has lower p*K*a values of (p*K*a1 = 8.19 and p*K*a2 = 6.99) when compared to chloroquine which are p*K*a1 = 10.03 and p*K*a2 = 7.94, respectively [51]. Ferroquine is ten-fold more concentrated at digestive vacuolar pH when compared to chloroquine. It is also 100-fold more lipophilic when compared to chloroquine at cytosolic pH. It targets the lipid site of hemozoin formation, forming a complex with haematin and inhibiting β-haematin formation [51,52]. Ferroquine also prevents the conversion of haematin into hemozoin by maintaining toxic haematin in an aqueous environment. In the oxidizing environment of the digestive vacuole of plasmodium parasites, it undergoes a reversible one-electron redox reaction with the generation of hydroxyl radicals, which affects its stability [51,52,53].

**Figure 17:**
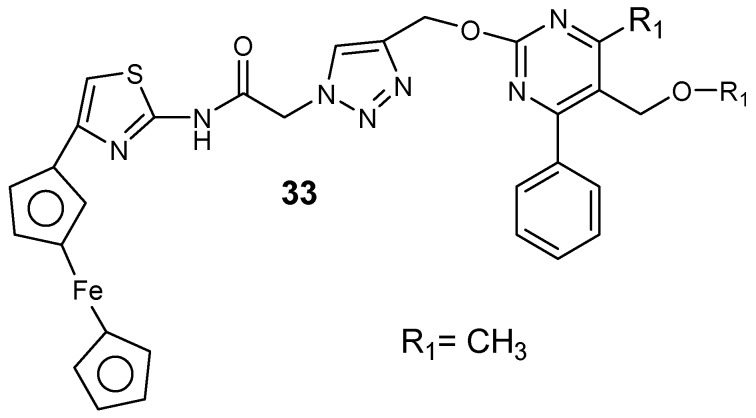
Structure of ferrocene–pyrimidine hybrid molecule **33**.

Chopra et al. synthesized ferrocene–pyrimidine hybrid molecules **33** (Figure 17) [54]. The presence of isopropyl and ethyl substituent at the C-5 position of the pyrimidine ring instead of the methyl group significantly enhanced the lipophilicity and antimalarial activity of the compounds. Hybrids containing an isopropyl ester group at the position with an aromatic substituent at position C-4 of the pyrimidine were more active when compared to the methyl and ethyl ester hybrid [54].

### 3.2. Polymer-Based Carriers for Antimalarial Drug Combination Therapies

#### 3.2.1. Polymer–Drug Conjugates

**Figure 18:**
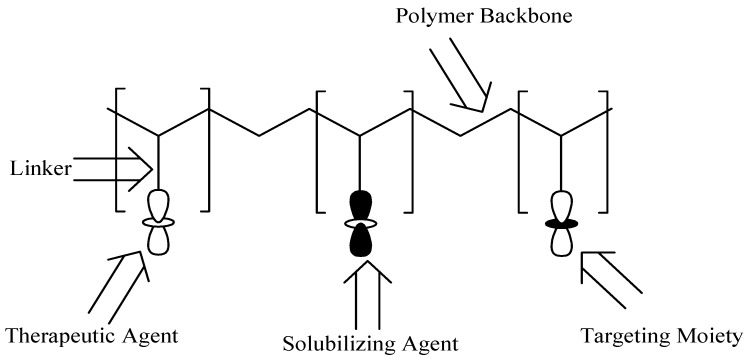
A schematic diagram of the Ringsdorf Model of polymer–drug conjugates.

Polymer–drug conjugates are polymer-based carriers that consist of three components: the drug, solubilizing agent, and targeting moiety. The polymer–drug conjugate model was suggested by Helmut Ringsdorf in 1975 (Figure 18) [55]. The three components are covalently linked via selected linkers, such as amines, esters, alcohols, and amides into the polymeric backbone [56]. The targeting moiety and solubilizing agent improve the therapeutic outcomes of the incorporated drug(s) into the polymers. There are several polymers that can be used for the formulation of these carriers such as polyaspartamides, polyglutamic acid, polyamidoamine carriers, etc. The advantages of polymer–drug conjugates include improved drug solubility, reduced drug toxicity, enhanced drug biodegradability and bioavailability [57], improved pharmacodynamics, pharmacokinetics, and pharmacological properties [58]. Polymer-based carriers can be utilized for combination therapy, which is very useful for the treatment of different diseases. Furthermore, they protect and preserve therapeutic agents during circulation from enzymatic attacks and they transport the bioactive agent to the target biological environment [59].

Kumar et al. explored combination therapy by synthesizing polymer–drug conjugates incorporated with dihydroartemisinin and primaquine utilizing substituted polyphosphazene carriers [60]. The antiplasmodial activity of these polymeric prodrugs was evaluated in vivo against *P. berghei* (NK65 resistant strains) utilizing diseased swiss albino mice. The conjugates displayed promising antiplasmodial activity at reduced dosage when compared to the standards of antimalarial drugs. The formulation of the polymer–drug conjugates to nanoparticles enhanced their uptake by the hepatocytes, resulting in targeted drug delivery. The incorporation of both drugs into the polymer was an effective approach which provided protection over a period of 35 days without any recrudescence, indicating their efficacy against resistant malaria. Aderibigbe and coworkers formulated polyaspartamide–drug conjugates incorporated with 4-aminoquinoline and ferrocene [61]. The drug release studies on the conjugates revealed sustained drug release at pH 7.4 and a fast drug release at pH 1.2. The sustained release mechanism of the drugs from the polymer backbone at pH 7.4 indicates that these conjugates are beneficial for combination therapy to combat the drug resistance which is common with the presently utilized antimalarials. The nature of the linker used for the incorporation of the drugs into the polymer influenced the mechanism of release of the drugs from the polymer carrier. The different release mechanisms of the incorporated drugs further revealed the potential of polymer–drug conjugates to overcome drug resistance [61].

Urban et al. designed polymer–drug conjugates by incorporating two standard antimalarial drugs chloroquine and primaquine into the poly(amidoamine) carriers [62]. These carriers exhibited high drug-loading capability. These conjugates were evaluated in vivo against *P. yoelii-*infected mice and they were active against *P. yoelii* in the infected erythrocytes [62]. In vitro and ex vivo evaluation indicated that the conjugates were highly selective, which was confirmed by their enhanced uptake into the *Plasmodium*-infected red blood cells when compared to the healthy red blood cells. Plasmodium-infected red blood cells are highly permeable to solutes with high molecular mass with distinct features such as polyelectrolyte behavior and the presence of amide groups in the main chain of the carriers. Merozoites invade red blood cells, resulting in the formation of a parasitophorous vacuole in which the parasite develops into rings, followed by trophozoites and schizonts. Schizont-infected red blood cells burst, releasing more merozoites which are responsible for the start of another blood cycle. The blood-stage infection is responsible for all symptoms and pathologies of malaria, making *Plasmodium*-infected red blood cells the main chemotherapeutic target. Presently, most antimalarial drugs do not have specificity for *Plasmodium*-infected red blood cells, resulting in the administration of antimalarial drugs at high dosage, leading to undesirable side effects and the development of resistant strains [62]. Employing polymer–drug conjugates is a potential approach to overcome drug resistance because the drugs are administered at low doses.

Aderibigbe at al. synthesized and evaluated in vitro polyaspartamide-based conjugates incorporated with two antimalarials, dihydrofolate reductase inhibitors [63]. The in vitro drug release profiles displayed controlled and slow drug release mechanisms. The most active conjugate incorporated with pyrimethamine and 4-aminosalicylic acid was active against *Plasmodium* parasite asexual stage with an IC_50_ value of 332.37 nM. In addition, the conjugate incorporated with pyrimethamine, primaquine, and 4-aminoquinolines displayed moderate antiplasmodial efficacy with an IC_50_ value of 4.71 nM [63]. Although polymer–drug conjugates with antimalarials have been reported to be active as antimalarials in vivo and in vitro, their mode of action is not fully understood. However, the controlled release mechanism of the drugs from the polymer and the capability of the polymer carrier to accommodate more than one drug molecule reveals the potential of polymer–drug conjugates to overcome drug resistance.

#### 3.2.2. Micelles and Dendrimers

**Figure 19:**
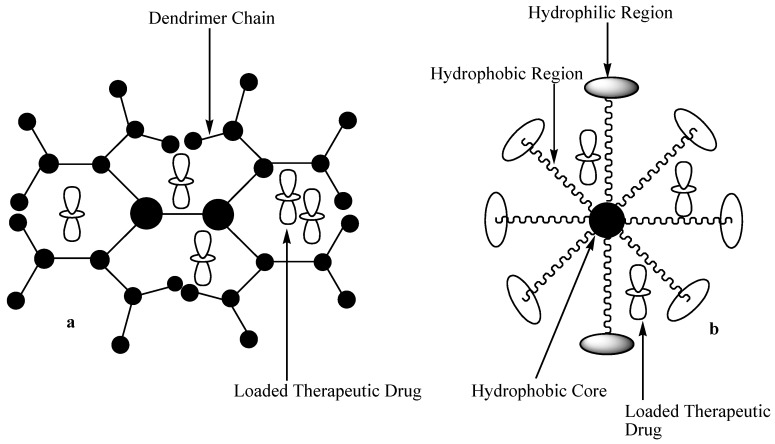
Schematic diagram showing (**a**) dendrimers loaded with drug and (**b**) micelles loaded with drug.

Polymeric micelles are nanocarriers (DDSs) made by self-assembling of surfactant molecules in aqueous solution with size ranging between 10 and 200 nm (Figure 19) [64]. These nanocarriers are characterized by long polymeric hydrophobic chains for the loading of the drugs and a hydrophilic head groups [65]. The advantages of micelles include controlled and targeted drug release, suitable for poorly soluble drugs, improve bioavailability, reduce drug toxicity, etc. [66]. On the other hand, dendrimers are polymeric nanocarriers that are highly branched, monodispersed, and have three-dimensional structures (Figure 17) [67]. These polymeric materials are very important in drug delivery due to their biocompatibility, low polydispersity index, and controlled molecular weight [68]. The functional group on the external layers of polymeric dendrimers are suitable for the encapsulation of therapeutic agents and the incorporation of a targeting moiety. Their intramolecular cavity is useful for the loading of therapeutic agents, resulting in improved drug activity, sustained drug release profile, and reduced drug toxicity [69].

Shi et al. designed polymeric micelles containing an antimalarial and an anticancer agent, chloroquine and docetaxel, for combination therapy [70]. The micelles, D-α-tocopheryl poly(ethylene glycol) and poly(ethylene oxide)-block-poly(propyleneoxide)-block-poly(e-caprolactone) were nanosized and exhibited controlled in vitro drug release. The hemolysis rate exhibited by these micelles was low, revealing their safety in vivo [70]. The combination of both drugs is a good approach that has the capability to suppress multidrug resistance. Codelivery of both drugs using polymeric micelles can reduce the amount of each drug administered, resulting in minimized toxic side effects.

Movellan et al. prepared dendrimers encapsulated with two standard antimalarial agents, primaquine and chloroquine using pluronic polymers and 2,2-bis(hydroxymethyl)propionic acid (bis-MPA) as polymers [71]. The formulated dendrimers loaded with antimalarial agents were screened in vitro against *P. falciparum* and against the mouse model of *P. yoelii* infection in vivo. These polymeric dendrimers displayed specific targeting to *Plasmodium*-infected erythrocytic cells when compared to the noninfected erythrocytes [71]. The specific targeting capability of the dendrimers to *Plasmodium*-infected red blood cells reveals the potential of dendrimers in the eradication of malarial infections.

#### 3.2.3. Hydrogels and in Situ Gels

**Figure 20:**
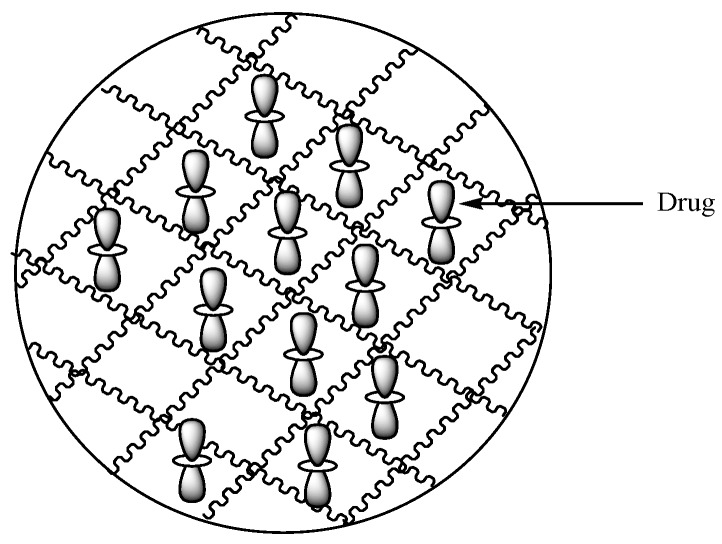
Schematic diagram showing hydrogels loaded with drugs.

Hydrogels are polymeric carriers with three-dimensional networks (Figure 20) formulated from synthetic and natural polymers. They have the capability to absorb and preserve large quantities of biological fluids and water. The degree of porosity of polymeric hydrogels is influenced by features such as the polymer composition, preparation method, and the materials from which they are derived, etc. There are several forms of hydrogels such as slabs, microparticles, films, and nanoparticles [72]. Hydrogels display unique advantages such as good biocompatibility, nonimmunogenicity, nontoxicity, environmental sensitivity (e.g., electric field, pH, and temperature), affordability, and their drug release mechanism can be tailored.

In situ gels are carriers that change into gels in the presence of biological environment or altered temperature and pH. The polymers that are employed for in situ gels formulations include carrageenan, guar gum, thiolated chitosan, pectin, and xanthan gum. In situ gels can be administered vaginally, intraperitoneally, rectally, nasally, or orally. These carriers display site specificity and reduce the negative effects of the loaded drugs [73].

Aderibgbe et al. synthesized gum acacia-based hydrogels loaded with curcumin and 4-aminoquinoline. The in vitro drug release profile revealed sustained and prolonged release of curcumin, whereby 4-aminoquinoline release was short-term at 37 °C. In addition, the preliminary studies revealed that hydrogels can be utilized for combination therapy for antiplasmodial drugs with various pharmacokinetics [74]. In another report, Aderibigbe et al. developed soy protein isolate-based hydrogels loaded with chloroquine and curcumin [75]. The hydrogels were pH sensitive, biodegradable, and displayed good swelling capability at pH 7.4. The release mechanisms of chloroquine and curcumin were influenced by the degree of crosslinking of the hydrogel and the presence of both drugs in the network. The release mechanisms of both drugs from the hydrogel network was a super case transport II, indicating the potential of using hydrogel for dual drug delivery of antimalarials in which the loaded drugs can work over different period of time with the ability to overcome drug resistance [75].

Dawre and coworkers designed polymeric in situ gels incorporated with artemether–lumefantrine-based combination therapy using poly(lactic-glycolic acid). The drug release studies were performed ex vivo and they exhibited sustained and controlled drug release mechanisms. These systems were safe for intramuscular administration. The formulation cured malaria without signs of recrudescence. The efficacy of in situ gels with a low dosage of the encapsulated drugs indicate that they are a potential strategy for the eradication of malaria [76].

#### 3.2.4. Nano- and Microcapsules

**Figure 21:**
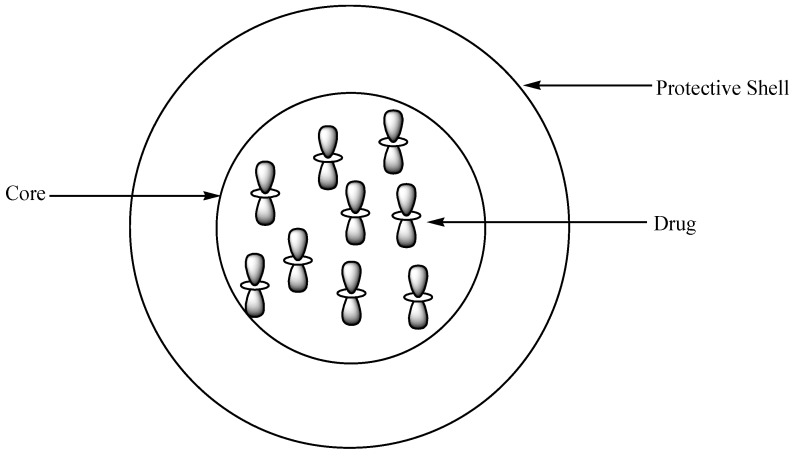
Schematic diagram showing polymer-encapsulated drug.

Polymeric capsules are drug delivery systems that are made up of a core and a protective shell where therapeutic agents are entrapped (Figure 21) [77]. There are several methods that are used for the preparation of polymer capsules, such as solidification of droplet shell, monoemulsion polymerization, self-assembly of block copolymers, etc. [78]. The capsules are categorized into two groups based on their size: nano- (10–1000 nm) and microcapsules (50 nm–2 mm) [79]. The advantages of polymer capsules include controlled and sustained drug release rate, reduced drug toxicity, high drug-loading capacity, improved drug bioavailability, and biodegradability [77].

Velasques et al. prepared and characterized nanosized polymer capsules incorporated with quinine and curcumin using polysorbate as the polymer. These capsules exhibited a diameter of 200 nm with slightly basic pH. The polymeric nanocapsules were active against *P. falciparum* in vitro when compared to the individual drugs, quinine and curcumin. The cytotoxicity evaluation revealed reduced toxicity of the loaded antimalarial agents in the capsules [80].

#### 3.2.5. Polymeric Nanoparticles

Polymeric nanoparticles are drug delivery systems that are solid colloidal particles or particulate dispersions with a diameter in the range of 1–1000 nm [81]. These polymer-based drug delivery vehicles are formulated from natural, synthetic, and semisynthetic polymers whereby the bioactive agent can be encapsulated, entrapped, loaded, dissolved, chemically incorporated, or absorbed [82]. Polymeric nanoparticles have interesting properties such as their good biodegradability, biocompatibility, and versatility in their application. The biodegradable polymers that are commonly utilized for the preparation of polymeric nanoparticles are poly(d,l-lactic-co-glycolic acid) (PLGA), polyalkylcyanoacrylates), poly(d,l-lactic acid), etc [83]. There are several advantages of polymeric nanoparticles, such as their targeted, sustained, and controlled drug release mechanism, improved water solubility, improved therapeutic efficacy of the bioactive molecule, and suitability for codelivery of drugs [84,85].

Jawahar et al. designed polymeric nanoparticles for combination therapy of clinically used antimalarial drugs, chloroquine, and the azalide antibiotic azithromycin, which has antiplasmodial efficacy, using PLGA polymer [85]. The in vitro antimalarial studies of PLGA nanoparticles incorporated with both drugs displayed synergistic effects against *Plasmodium* parasite growth with an IC_50_ value of 1.11 µg/mL and EC_50_ of 1.95 µg/mL. The nanoparticles exhibited a particle size of 89.6 nm, polydispersity index of 0.24, zeta potential of −13.2 mV, and the in vitro evaluation revealed that codelivery of chloroquine and azithromycin is capable of overcoming drug resistance via intracellular targeting [85]. Anand et al. formulated β-cyclodextrin polymer nanoparticles loaded with artemisinin and doxorubicin. Their photophysical and spectroscopic properties were investigated using fluorescence, UV-vis absorption, and circular dichroism in neutral aqueous media. The nanoparticles’ capability to disrupt the DOX dimers in solution was significant. The nanoparticles were able to load artemisinin deeply into the nanoparticle frame. The nanoparticles were found to be suitable for sustained and controlled drug release and improved drug bioavailability. The spectroscopic data revealed the alcohol-like character of the artemisinin environment and the photophysical parameters indicated the inherent emission capability of DOX in the hydrophobic interior of the polymer nanoparticles [86]. Furthermore, Oyeyemi and coworkers designed polymeric nanoparticles for codelivery of artesunate and curcumin, utilizing PLGA as a polymer via an oil-in-water single emulsion method [87]. These nanoparticles were characterized for the polydispersity index (PDI), zeta potential, particle size, and entrapment efficiency; and their antimalarial activity was evaluated in vivo against *P. berghei* mice model at doses of 5 and 10 mg/kg. The zeta potential and PDI of the polymeric nanoparticle were −19.1 and 0.141 mV, respectively. The particle size of the artesunate–curcumin-loaded nanoparticles was 251 nm, while drug entrapment efficiency was 22.3%. The drug-release profile of the formulation was controlled and sustained over a period of seven days. The percentage destruction of *P. berghei* was significant in 5 mg/kg artesunate–curcumin-loaded PLGA at day 5 (79.0%) and at day 8 (72.5%) when compared to the free drug [87]. The release of curcumin and artesunate was prolonged for more than a week in vitro, a unique feature capable of improving the antimalarial activity over a prolonged period. The nanoparticles were able to deliver the encapsulated drugs to target organs, indicating the formulation’s ability to target the parasite schizonts in the liver or spleen. The formulation was nontoxic with low enzyme indicators of hepatoxicity. The formulation was effective against malaria parasites at a low concentration [87].

#### 3.2.6. Liposomes

**Figure 22:**
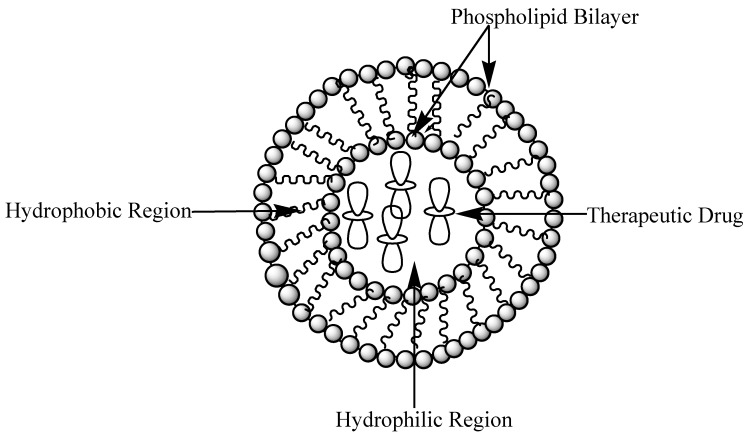
Schematic diagram of a liposome loaded with drug.

Liposomes are small and artificial spherical-shaped vesicles. They are prepared from phospholipids and cholesterol. They exhibit hydrophilic and hydrophobic properties with good biocompatibility (Figure 22) [88]. Their properties differ in terms of size, surface charge, composition of lipids, and method of preparation [89]. The nature of their bilayers, such as rigidity, permeability, and bilayer charge, is influenced by the components used to prepare them [90]. Their particle sizes are in the range of 30 nm to several micrometers. They are composed of lipid bilayers surrounded by aqueous units, in which the polar head groups are oriented in the pathway of the exterior and interior aqueous phases [91]. Liposomes are employed as carriers for hydrophilic or hydrophobic drug molecules [90,92]. Their low toxicity, biodegradability, biocompatibility, and capability to encapsulate lipophilic and hydrophilic drugs [93] and facilitate site-specific drug delivery makes them useful for the delivery of antimalarials [90].

Ibrahim et al. loaded trans platinum-chloroquine diphosphate dichloride into liposomes as a potential antiplasmodial drug-delivery system [94]. The therapeutic agent was loaded into the interior of the cationic liposome via the thin drug–lipid film method. The PEGylated cationic liposomes and neutral liposomes showed minimum leakage of drugs after two months of storage at 4 °C, and further showed a slow release in vitro at 37 °C for 72 h.

Aditya et al. prepared curcminoid-loaded liposomes by a thin-film hydration method and combined them with α/β arteether [95]. The formulation was administered intravenously and evaluated in *Plasmodium berghei*-infected mice. The combination therapy of curcuminoid-loaded liposomes (40 mg/kg body wt) along with α/β arteether (30 mg/kg body wt) cured the infected mice and prevented recrudescence significantly when compared to the liposome formulation alone [95]. Rajendran et al. prepared liposomes for the delivery of monensin in combination with artemisinin [96]. The formulation was prepared from soya phosphatidylcholine, cholesterol, and distearoyl phosphatidylethanolamine-methoxy-polyethylene glycol 2000. The effect of the combination against *Plasmodium falciparum* (3D7) cultures and mice models infected with *Plasmodium berghei* strains ANKA and NK65 resulted in enhanced killing of the parasites, inhibited parasite recrudescence, and enhanced survival [96]. The effect of the combination was significant at the trophozoite and schizont stages when compared to the ring stage of the parasite. The intracellular delivery of liposomal drug loaded formulation at a high concentration to parasitophorous vacuoles of infected erythrocytes is due to the significant antiplasmodial action when used in combination with the free artemisinin. This finding indicates that liposome formulation with artemisinin can overcome drug resistance in *P. falciparum* and prevent malaria relapse [96]. The enhanced uptake of liposomes in brain tissue further suggests that the formulation is effective in treating cerebral malaria during *P. falciparum* infection.

Liposomes can overcome drug-resistant malaria because of their capability to bypass the chloroquine transponder and transportation via cell membrane by mechanisms such as membrane fusion or entrapment of chloroquine in pH-sensitive liposomes [90]. However, their high cost of production, cytotoxic effects, and complications in sterilization and storage limit their use in drug delivery.

## 4. Conclusions

In this review article, two combination therapy strategies for the eradication of malaria were discussed as potential approaches that can overcome drug resistance that is common with the presently employed antimalarial drugs. Most of the hybrid compounds were reported to be effective against *Plasmodium* parasite when compared to the individual parent drugs. The design of the hybrid compounds and the nature of the linker between the hybridized molecules plays a significant role in the antimalarial activity of the compounds. In addition, some of the hybrid compounds show cytotoxic effects on mammalian cells.

The polymer-based carriers containing antimalarials displayed good therapeutic outcomes when compared to the hybrid compounds. However, most of them have not yet reached clinical trials. The reported therapeutic outcomes, such as improved drug hydrophilicity, targeted drug delivery, protection of drug activity in a biological environment, and sustained and controlled drug release, suggest that employing polymer-based carriers for the delivery of antimalarials is a potential approach which must be explored. There are very few reports on polymer-based carriers containing two or more antimalarial drugs when compared to hybrid compounds. There is an urgent need for researchers to develop more polymer-based carriers containing antimalarials. Continual research on the polymer-based carriers and hybrid molecules for the delivery of antimalarials will result in the discovery and design of potent therapeutics with excellent antimalarial activity.
